# Elevated Interleukin-18 Receptor Accessory Protein Mediates Enhancement in Reactive Oxygen Species Production in Neutrophils of Systemic Lupus Erythematosus Patients

**DOI:** 10.3390/cells10050964

**Published:** 2021-04-21

**Authors:** Jie Ma, Ian Kar Yin Lam, Chak-Sing Lau, Vera Sau Fong Chan

**Affiliations:** Division of Rheumatology and Clinical Immunology, Department of Medicine, Li Ka Shing Faculty of Medicine, The University of Hong Kong, Hong Kong SAR, China; jiema@hku.hk (J.M.); ianlam8816@gmail.com (I.K.Y.L.); cslau@hku.hk (C.-S.L.)

**Keywords:** SLE, interleukin-18 receptor accessory protein, type I interferon, cellular function

## Abstract

Interleukin-18 receptor accessory protein (IL18RAP) is an indispensable subunit for the IL-18 receptor (IL-18R) complex’s ability to mediate high-affinity IL-18 binding and signalling transduction. Interest in IL-18 in systemic lupus erythematosus (SLE) has been mostly focused on its role as a type 1 T helper cell-driving cytokine. The functional significance of IL18RAP in mediating the IL-18-driven response in myeloid cells in SLE remains largely unexplored. This study aimed to investigate the expression and function significance of IL18RAP in neutrophils of SLE patients. By qRT-PCR and Western blot analyses, elevated expressions of IL18RAP mRNA and protein were observed in neutrophils from SLE patients—particularly those with a history of nephritis. IL18RAP expression correlated negatively with complement 3 level and positively with disease activity, with higher expression in patients exhibiting renal and immunological manifestations. The increased IL18RAP expression in SLE neutrophils could be attributed to elevated type I interferon level in sera. Functionally, neutrophils from SLE patients showed higher IL-18-mediated enhancement in reactive oxygen species (ROS) generation, which showed positive correlation with IL18RAP expression and could be neutralized by anti-IL18RAP blocking antibodies. Taken together, our findings suggest that IL-18 could contribute to SLE pathogenesis through mediation of neutrophil dysfunction via the upregulation of IL18RAP expression.

## 1. Introduction

Systemic lupus erythematosus (SLE) is a female-biased, chronic systemic autoimmune disorder that affects multiple organ systems, including renal, nervous, musculoskeletal, haematological, respiratory, and cardiovascular systems [1,2]. Renal involvement is one of the most common clinical features of SLE, with approximately 50–60% of Asian SLE patients developing lupus nephritis (LN) during the disease course. It is also a major cause of morbidity and mortality in SLE patients [3,4]. SLE pathogenesis is highly complex; the intricate interplay of environmental, genetic, and hormonal factors lead to disturbances in both the innate and adaptive immune compartments and the loss of self-tolerance [5]. Cytokine dysregulation is a typical characteristic of SLE. Imbalanced proinflammatory and anti-inflammatory cytokine production, together with abnormal responsiveness of immune cells to various cytokines, exacerbate immune dysregulation and contribute to tissue inflammation and organ damage in lupus [6].

Interleukin-18 (IL-18) is a proinflammatory cytokine, also known as the inducing factor for interferon-gamma (IFN-γ). Its receptor (IL-18R) consists of two subunits that belong to the interleukin-1 receptor (IL-1R) family. The alpha chain (IL-18Rα or IL18R1) has a weak binding affinity for IL-18. Upon binding of IL-18 to IL-18 Rα, the other subunit—the IL-18 receptor accessory protein (IL18RAP, also known as IL-18Rβ)—is then recruited to form a heterotrimeric complex for high-affinity binding [7,8]. IL-18 stimulation then initiates cell signalling through recruitment of the myeloid differentiation primary response protein 88 (MyD88) via the toll-interleukin-1 receptor (TIR) domain of the IL-18R, thereby activating the nuclear factor kappa-light-chain-enhancer of activated B cells (NF-κB) and mitogen-activated protein kinase (MAPK) pathways [9,10]. Accumulative evidence from animal models, as well as clinical studies, suggests that IL-18 activity plays a pathological role in the development of SLE. Elevated circulating levels of IL-18 were reported in both paediatric-onset and adult SLE patients with positive correlation to disease activity [11,12,13]. In particular, it has been shown to associate with LN development. Elevated levels of IL-18 were observed in kidney biopsies, as well as in serum of LN patients, when compared with lupus patients without nephritis [13,14,15]. In addition, a high basal serum IL-18 level was shown to be associated with active renal disease [16]. Similarly, increased IL-18 levels were also found in serum and nephritic kidneys of MRL/*lpr* lupus mice [17,18]. In this lupus mouse model, a continued daily administration of IL-18 resulted in renal damage exacerbation as characterized by proteinuria, glomerulonephritis, and vasculitis [18]; whereas in vivo neutralization of IL-18 attenuated glomerulonephritis and even prolonged animal survival [19], suggesting that IL-18 signalling is important for lupus and LN development. Indeed, IL-18Rα-deficient MRL/*lpr* mice exhibited decreased levels of proteinuria and serum anti-DNA antibodies, attenuation in renal pathology, and longer survival [20]. In addition, lymphocytes of MRL/*lpr* mice were found to have an overexpression of *Il18rap*, which could contribute to the heightened response to IL-18 and higher levels of IFN-γ production [21]. In fact, interest in the pathological role of IL-18 in SLE is mostly focused on its effect on T cells as a type 1 T helper cell (Th-1)-driving cytokine [22].

Apart from T cells, IL-18 also activates natural killer (NK) cells and neutrophils [23,24,25]. In a screening assay, we observed an elevated expression of IL18RAP in peripheral leukocytes of LN patients and its expression level was highly correlated with neutrophil-associated genes (Figure S1). So far, little is known on the role of IL18RAP in SLE. In this study, by expression analyses and in vitro cell culture assays, we tested whether IL18RAP has any functional significance in neutrophils of SLE patients.

## 2. Materials and Methods

### 2.1. Human Subjects

SLE patients fulfilling both the 1997 American College of Rheumatology (ACR) and the 2012 Systemic Lupus International Collaborating Clinics (SLICC) criteria were recruited from the Queen Mary Hospital, Hong Kong. LN patients were SLE patients with biopsy-confirmed nephritis. Non-nephritic lupus (NLN) patients were SLE patients with no history of renal manifestation for at least ten years after disease onset. Age- and sex-matched healthy control (HC) samples were collected from buffy coats obtained from healthy blood donors from the Hong Kong Red Cross. All participants gave their informed consent for inclusion in the study. The study was approved by the Institutional Review Board of the University of Hong Kong/Hospital Authority Hong Kong West Cluster (study approval number: UW 16-286).

### 2.2. Isolation and Stimulation of Human Neutrophils

Neutrophils were isolated from buffy coats or fresh EDTA-anticoagulated blood. Reagents used in the isolation and culture process were tested to contain <0.5 EU/mL endotoxin level using a Pierce Chromogenic Endotoxin Quant Kit (Thermo Fisher Scientific, Rockford, IL, USA). Briefly, neutrophils were separated using Ficoll-Paque Plus (GE Healthcare Life Science, Uppsala, Sweden) gradient centrifugation, followed by erythrocyte sedimentation in 6% dextran (Sigma-Aldrich, Oslo, Norway). Remaining erythrocytes were lysed with deionized water for 30 s, followed by neutralization with PBS. Neutrophils were cultured in RPMI 1640 medium supplemented with 20 mM GlutaMAX, 1 mM sodium pyruvate, nonessential amino acids, 100 U/mL penicillin, 100 μg/mL streptomycin, and 10% foetal bovine serum (all from Gibco, Gaithersburg, MD, USA). For stimulation, purified neutrophils were treated with or without 1000 IU/mL recombinant human interferon-alpha (IFN-α) (Abcam, Cambridge, UK) at 37 °C for 6 h in specified assays. In separate experiments, neutrophils were incubated with 10% human sera derived from healthy controls or from SLE patients with low or high interferon (IFN) activity. Type I IFN activity in individual serum samples was determined using HEK-Blue IFNα/β cells and a QUANTI-Blue assay system (InvivoGen, San Diego, CA, USA). SLE sera with high levels of IFN (IFN^hi^) were pooled from sera with more than 500 IU/mL, whereas IFN^lo^ sera were pooled from sera with less than 100 IU/mL.

### 2.3. Real-Time Quantitative Reverse Transcription Polymerase Chain Reaction (qRT-PCR)

Cells were lysed in TRI Reagent (Molecular Research Center Inc., Cincinnati, OH, USA), and RNA was separated according to the manufacturer’s recommendation using 1-bromo-3-chloropropane (Molecular Research Center Inc., Cincinnati, OH, USA) and precipitated by isopropanol (Sigma-Aldrich, St. Louis, MO, USA). The overall quality of RNA was assessed by agarose gel electrophoresis. RNA was reverse transcribed into cDNA using a PrimeScript RT Reagent Kit (Takara, Kusatsu, Japan). Quantitative real-time PCR was performed using a StepOnePlus Real-Time PCR System (Applied Biosystems, Foster City, CA, USA) with SYBR Premix Ex Taq (Tli RNaseH Plus) (Takara, Kusatsu, Japan) under the following cycling conditions: 20 s at 95 °C, 20 s at 62 °C, and 20 s at 72 °C for 40 cycles. Gene expression was normalized with glyceraldehyde-3-phosphate dehydrogenase (GAPDH) expression and expressed as relative quantity (RQ), calculated by the formula (threshold cycle method): 2^−[ΔCt (test sample)−ΔCt (reference sample)].^ PCR primer sequences were as follows: human GAPDH (NM_002046.7), forward 5′–TTC ACC ACC ATG GAG AAG GC–3′ and reverse 5′–GAT GGC ATG GAC TGT GGT CA–3′; human IL18RAP (NM_003853.3), forward 5′–TGA AGA ACA CTT GGC CCT GA–3′ and reverse 5′–GCA AGA TTC ACT GCT GCT TGT–3′; human interferon-induced protein with tetratricopeptide repeats 1 (IFIT1) (NM_001548.5), forward 5′–GCA GGC TGT CCG CTT AAA TC–3′ and reverse 5′–CCA CAG AGC CTT TTC TTC GG–3′. All primers were qPCR-validated to generate single-sized products. 

### 2.4. Western Blotting

Whole-cell lysate was obtained by lysing neutrophils with lysis buffer (Cell Signaling Technology, Danvers, MA, USA) of 20 mM Tris-HCl, pH 7.5, 150 mM NaCl, 1 mM Na_2_EDTA, 1 mM EGTA, 1% Triton, 2.5 mM sodium pyrophosphate, 1 mM beta-glycerophosphate, 1 mM Na_3_VO_4_, 1 μg/mL leupeptin, 1 mM PMSF (Sigma-Aldrich, Steinheim, Germany) and protease inhibitor cocktail. Protein from each sample (30 μg) was separated by SDS-PAGE and transferred to PVDF membrane (GE Healthcare Life Science, Freiburg, Germany), followed by incubation with anti-IL18RAP monoclonal antibody (clone 290, Invitrogen, Rockford, IL, USA) or with anti-β-actin antibody (clone AC-74, Sigma-Aldrich, St. Louis, MO, USA) overnight at 4 °C. After incubation with HRP-conjugated antirabbit or antimouse IgG (Santa Cruz, Dallas, TX, USA), membranes were exposed to enhanced chemiluminescence substrate. Signals were detected by a ChemiDoc MP Imaging System (Bio-Rad Laboratories, Hercules, CA, USA). Densitometry reading of the blots was analysed by Image Lab Software (Bio-Rad Laboratories, Hercules, CA, USA) and IL18RAP signal intensity was normalized with the β-actin level in the corresponding sample.

### 2.5. Measurement of Reactive Oxygen Species (ROS)

Freshly isolated neutrophils were dispensed in triplicate into a 96-well microplate, pretreated with or without 100 ng/mL recombinant human IL-18 (rhIL-18) (InvivoGen, San Diego, CA, USA) for 2 h at 37 °C. Then, 1 μM dihydrorhodamine 123 (DHR123) (Invitrogen, Eugene, OR, USA) was added to the wells for 15 min before stimulation with 100 nM N-formyl-methionyl-leucyl-phenylalanine (fMLP) (Sigma-Aldrich, St. Louis, MO, USA). For the blocking assay, 10 μg/mL polyclonal anti-IL18RAP antibody (Invitrogen, Rockford, IL, USA) or goat IgG isotype control (Novus Biologicals, Centennial, CO, USA) was incubated with neutrophils for 20 min at 37 °C prior to stimulation with rhIL-18, DHR123, and fMLP, as described above. Fluorescence signal (FS) was detected in real time at 529 nm and recorded at 2- to 5-min intervals for 30 min using a CLARIOstar microplate reader (BMG Labtech, Ortenberg, Germany). Enhancement in fMLP-mediated ROS generation at 30 min was calculated using the formula: (FS[rhIL18+fMLP]−FS[fMLP])/FS[fMLP] × 100%.

### 2.6. Statistical Analyses

Data were analysed using GraphPad Prism 8 software (GraphPad Software Inc., San Diego, CA, USA). Normal distribution of the data was verified by the Shapiro–Wilk normality test prior to statistical analysis. Statistical significance between two groups was calculated using the Mann–Whitney *U* test. A paired *t* test was used to compare two different treatments that were applied to the same samples. Correlation analysis was performed by Spearman’s rank-order correlation test. All *p*-values were considered significant at *p* < 0.05.

## 3. Results

### 3.1. Neutrophils of SLE Patients Show Elevated IL18RAP Expression, Which Correlates Positively with Disease Activity and Renal Involvement

**Figure 1:**
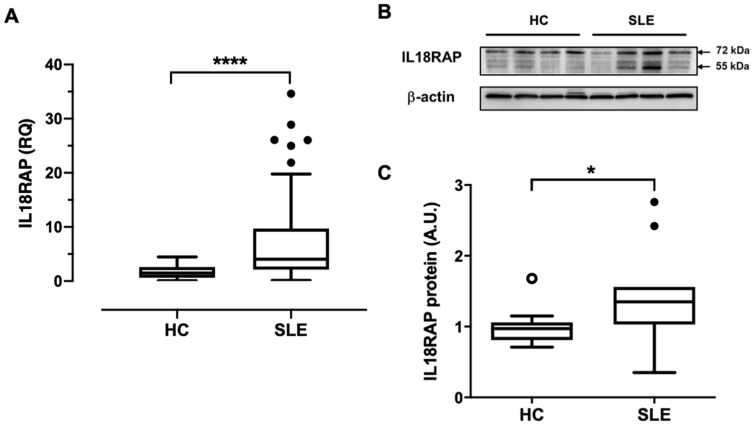
Elevated expression of IL18RAP in neutrophils from SLE patients. (**A**) qRT-PCR analysis on IL18RAP expression in neutrophils from healthy controls (HC, *n* = 30) and SLE patients (*n* = 95). RQ, relative quantity; **** *p* < 0.0001 by Mann–Whitney *U* test. (**B**) Western blotting on protein expression of IL18RAP in neutrophils was performed using an independent set of samples. Representative blot of three independent experiments showing four samples in HC and SLE patients. Arrows indicate the position of molecular weight markers. (**C**) Summary chat showing relative IL18RAP protein expression in neutrophils between HC (*n* = 11) and SLE patients (*n* = 11), as measured by normalized densitometry signal intensity. A.U., arbitrary unit; * *p* < 0.05 by Mann–Whitney *U* test. Data were shown as the median (horizontal line) with interquartile range (IQR, box), lower and upper whiskers (data within 1.5 × IQR), and outliers (points) (Tukey’s box).

Results from our pilot screening assay on SLE peripheral leukocytes showed highly correlated expression between IL18RAP and neutrophil-associated genes, and the former was significantly elevated in patients with nephritis development (Figure S1). We therefore hypothesized that IL18RAP in neutrophils could be dysregulated in expression and function in lupus patients. To investigate this, we first compared the expression of IL18RAP in neutrophils of HC (*n* = 30) and SLE patients (*n* = 95). qRT-PCR analysis indeed showed higher mRNA expression of IL18RAP in SLE patients (Figure 1A). At the protein level, an independent set of HC (*n* = 11) and SLE (*n* = 11) samples were found to express the canonical isoform of IL18RAP protein at the expected molecular weight of around 68 kDa by western blotting (Figure 1B). However, in some samples, significant immunoreactivity was also detected at a lower molecular weight, which likely corresponds to the shorter IL18RAP isoform with approximately 52 kDa (UniProtKB identifier: O95256-2). Nevertheless, an increased expression of IL18RAP protein was observed in neutrophils from SLE patients (Figure 1B,C). These results indicate that IL18RAP expression is dysregulated in SLE neutrophils.

**Figure 2:**
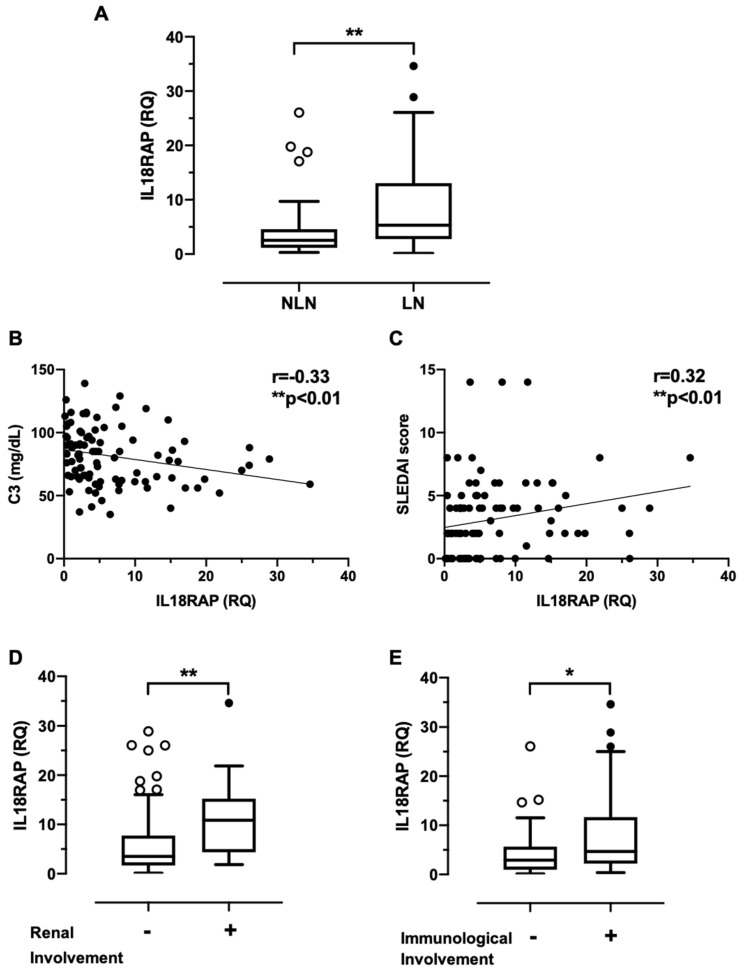
Correlation analyses between IL18RAP expression and clinical and laboratory parameters in 95 SLE patients. (**A**) IL18RAP expression was compared between non-nephritic lupus patients (NLN, *n* = 40) and lupus nephritis patients (LN, *n* = 55). ** *p* < 0.01 by Mann–Whitney *U* test. Data were expressed as Tukey’s boxes. Scatter plots showing IL18RAP expression correlation with (**B**) C3 levels, and (**C**) SLEDAI-2K scores in SLE patients (*n* = 95). Linear regression line is shown; r = correlation coefficient; ** *p* < 0.01 by Spearman’s rank-order test. Stratified analyses on IL18RAP expression comparison between SLE patients (**D**) with (+, *n* = 12) and without (−, *n* = 83) renal involvement; and between SLE patients (**E**) with (+, *n* = 64) and without (−, *n* = 31) immunological involvement at the time of blood sample collection. * *p* < 0.05; ** *p* < 0.01 by Mann–Whitney *U* test (for **A**,**D**,**E**). Results were shown as Tukey’s boxes. RQ, relative quantity.

We then examined if the elevated expression of IL18RAP had any association with clinical manifestations in these 95 SLE patients. Basic demographics and selected clinical characteristics of the study participants were shown in Table 1. As IL-18 has been implicated in nephritis development, we specifically compared SLE patients with biopsy-confirmed nephritis (LN group, *n* = 55) with patients without any renal manifestations for at least 10 years (non-LN, NLN group, *n* = 40). Reinforcing our pilot data, LN patients indeed expressed significantly higher IL18RAP when compared with NLN patients (Figure 2A). Also, the expression level of IL18RAP correlated negatively with serum complement 3 (C3) level, and positively with SLE disease activity index 2000 (SLEDAI-2K) score (Figure 2B,C). No significant correlation was observed with other serological parameters such as anti-dsDNA antibody, C4, urea, creatinine, albumin, or globulin levels. We further investigated if the dysregulated IL18RAP expression was associated with specific organ or system manifestations. As shown in Figure 2D,E, higher IL18RAP expression was observed in SLE patients exhibiting active manifestations of the renal system (presence of urinary casts, haematuria, proteinuria, or pyuria—as defined by the descriptors in the SLEDAI-2K scoring system) and the immunological system (low complement or increased DNA binding).

### 3.2. IL18RAP Expression in Neutrophils Is Regulated by Type I Interferon

**Figure 3:**
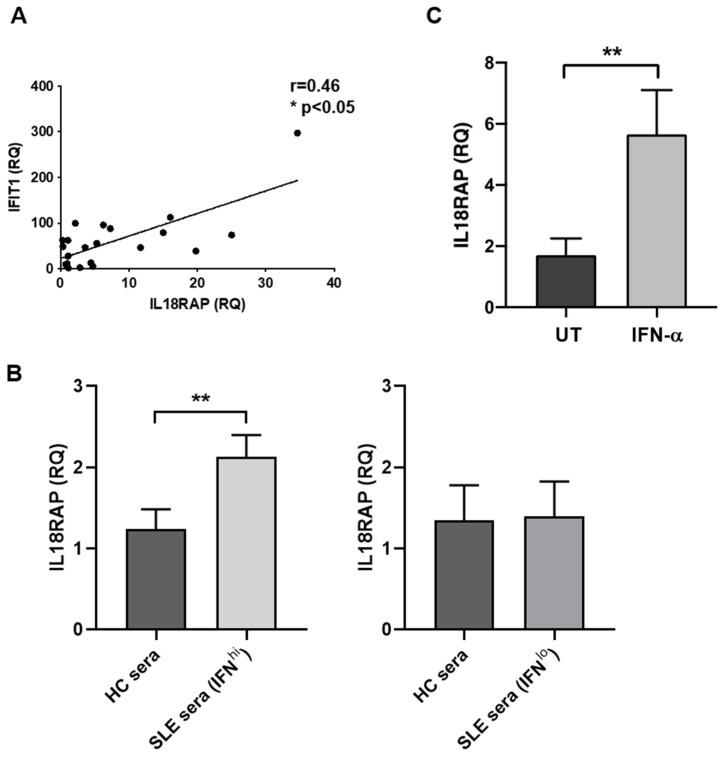
Regulation of IL18RAP expression by type I interferon (IFN) in neutrophils. (**A**) Expression correlation between IL18RAP and IFIT1 in neutrophils of SLE patients (*n* = 21). Linear regression line is shown; r = correlation coefficient; * *p* < 0.05 by Spearman’s rank-order test. (**B**) IL18RAP expression in healthy neutrophils upon culture with sera from HC and SLE patients was evaluated. Samples from healthy individuals (*n* = 12) were treated with HC sera and SLE sera with high IFN activity (IFN^hi^) (left panel). Six individuals (*n* = 6) were treated with HC sera and SLE sera with low IFN activity (IFN^lo^) (right panel). A paired *t* test was used to compare data from the same samples treated with HC sera and SLE sera. (**C**) IL18RAP expression in healthy neutrophils (*n* = 8) was compared between those cultured in medium alone (untreated group, UT) and those cultured in the presence of recombinant human IFN-α (1000 IU/mL for 6 h). ** *p* < 0.01 by paired *t* test. RQ, relative quantity. Data are shown as mean + SEM.

Next, we asked whether the inflammatory milieu in serum could have an impact on IL18RAP expression in SLE patients. SLE peripheral leukocytes remarkably express a spectrum of IFN signature genes as a result of perturbed production and signalling of type I IFN [26]. We therefore hypothesized and tested a causal relationship between type I IFN and IL18RAP using separate sets of HC and SLE samples. Firstly, a positive expression correlation between IL18RAP and IFIT1, one of the interferon-stimulated genes (ISGs), was observed (Figure 3A), suggesting that IL18RAP expression in SLE neutrophils might be regulated by IFN in circulating blood. Next, sera from HC and SLE patients were tested for the capacity to modulate IL18RAP expression in neutrophils. As shown in Figure 3B, only SLE sera with high level of type I IFN, but not those with low IFN, could upregulate IL18RAP expression when compared with cells cultured in HC sera. Furthermore, an increase in IL18RAP expression could be readily induced upon recombinant IFN-α stimulation in vitro (Figure 3C). Taken together, it is likely that type I IFN in circulating blood could cause, at least in part, the upregulated IL18RAP expression in neutrophils of SLE patients.

### 3.3. IL-18 Enhances fMLP-Mediated ROS Generation in SLE Neutrophils

**Figure 4:**
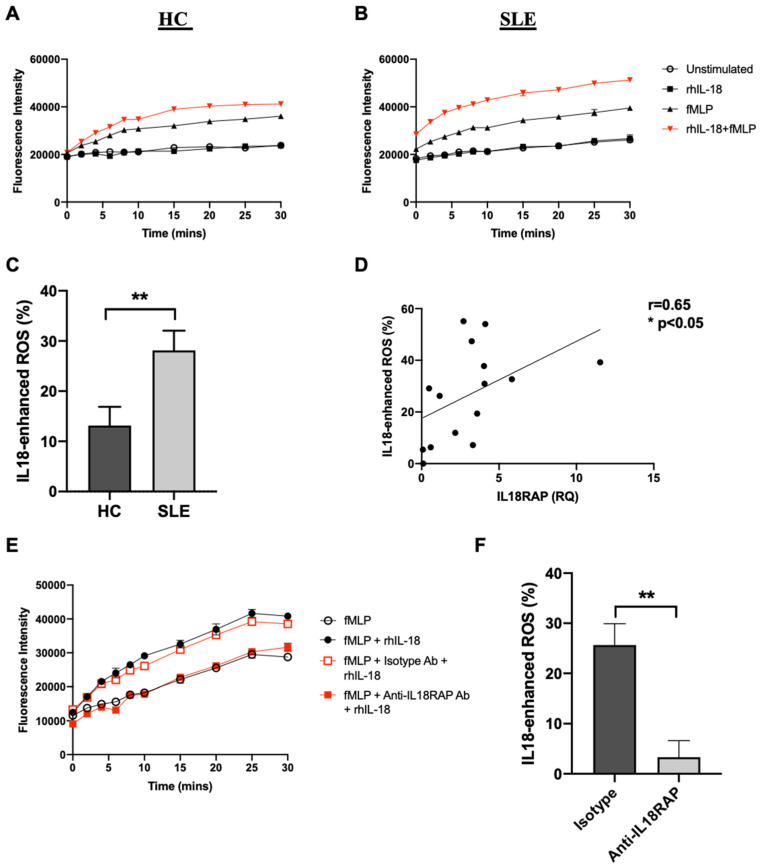
IL-18 enhances fMLP-mediated ROS generation in neutrophils via IL18RAP. Representative plots showing kinetics of ROS generation in neutrophils from (**A**) HC and (**B**) SLE patients upon treatment with or without rhIL-18 (100 ng/mL) and fMLP (100 nM) stimulation. (**C**) Summary chart showing IL-18-enhanced ROS generation in neutrophils at 30 min poststimulation in HC (*n* = 14) and SLE patients (*n* = 14). ** *p* < 0.01 by Mann–Whitney *U* test. (**D**) Correlation analysis between IL18RAP expression and IL-18-enhanced ROS generation using an independent set of HC and SLE neutrophil samples (*n* = 15). * *p* < 0.05 by Spearman’s rank-order correlation test; r = correlation coefficient and the linear regression line is shown. (**E**) Representative plot showing fMLP-mediated ROS generation in SLE neutrophils with anti-IL18RAP antibody (Ab) or goat IgG isotype control (10 μg/mL) pretreatment prior to IL-18 (100 ng/mL) stimulation. (**F**) Summary chart showing IL-18-enhanced ROS generation in SLE neutrophils (*n* = 3) upon pretreatment with goat IgG isotype antibody or anti-L18RAP antibody at 30 min poststimulation. ** *p* < 0.05 by paired *t* test. Data shown as mean + SEM.

We then assessed the functional impact of the elevated IL18RAP expression in SLE neutrophils. The inflammatory and antimicrobial action of neutrophils is highly associated with the induction of a strong oxidative burst, generating almost the whole spectrum of ROS including superoxide, hydrogen peroxide, and hypochlorous acid [27]. N-formyl-methionyl-leucyl-phenylalanine (fMLP), a bacterial analogue, is one of the most common agents used to study ROS generation in research. We therefore used fMLP to evaluate neutrophil functional response. Our results showed that IL-18 stimulation alone had no effect on the induction of ROS generation in neutrophils from both HC and SLE patients, however, it could augment fMLP-mediated ROS generation in vitro (Figure 4A,B). Notably, the level of IL-18-mediated ROS enhancement was significantly higher in neutrophils from SLE patients than in those from HC (Figure 4C). Interestingly, this enhancement positively correlated with the expression level of IL18RAP (Figure 4D). Although neutrophils from HC and SLE were obtained from samples of slightly different preparation—i.e., buffy coat and fresh blood, respectively—it was anticipated to have minimal impact on the functional response of neutrophils, including oxidative burst, phagocytosis, migration, and formation of neutrophil extracellular traps (NETs) [28,29]. Nevertheless, we compared HC neutrophils from fresh blood and buffy coat, and observed that they indeed had similar ability to produce ROS in vitro (Figure S2). We also evaluated the expression of the IL-18Rα subunit in neutrophils and found no significant difference between HC and SLE patients (Figure S3). Thus, it is likely that the augmentation in IL-18-mediated ROS enhancement in SLE neutrophils is attributable to the dysregulation of IL18RAP expression. In support of this argument, IL-18-mediated enhancement in ROS generation in SLE neutrophils could indeed be efficiently neutralized by anti-IL18RAP antibody (Figure 4E,F). Finally, we further tested whether IL-18-mediated ROS enhancement in neutrophils could be observed using an SLE-relevant stimulus—the anti-dsDNA immune complexes. Similar to fMLP stimulation, immune complex-induced ROS was also increased in response to IL-18 pretreatment, especially in neutrophils from SLE patients (Figure S4). Taken together, our data suggests that heightened IL18RAP expression may contribute to the dysregulated function of neutrophils in SLE patients.

## 4. Discussion

IL18RAP is an indispensable subunit of the IL-18R complex for the mediation of high-affinity IL-18 binding and subsequent signalling transduction [8]. Its extracellular region contains two immunoglobulin-like domains for ligand interaction, and the cytoplasmic tail possesses a TIR domain which is also found in IL-1R and many toll-like receptors (TLRs). In mice, IL18RAP deficiency led to pronounced impairment in IL-18-induced responses in multiple cell types, including IFN-γ production in Th-1 cells, NK cell cytotoxicity, as well as neutrophil activation [30]. Also, responses to IL-1 and TLR ligand stimulation remained normal in these mice, indicating that this receptor is specific for mediating the IL-18 signalling cascade. The majority of studies on IL18RAP focus on genetic association analyses that encompass a diverse spectrum of conditions including cancer, cardiovascular disease, autoimmunity, and infections. Pertaining to inflammatory disorders, the *rs917997* single nucleotide polymorphism (SNP) in *IL18RAP* was found to have a divergent role, conferring risk for celiac disease but protection for type I diabetes [31]. A follow-up study further demonstrated that the risk-associated allele of this SNP could yield a higher IFN-γ production in peripheral blood mononuclear cells (PBMCs) upon IL-12 and IL-18 costimulation [32]. Using combined genome-wide association studies (GWAS) and expression quantitative trait loci (eQTL) analyses, Andiappan et al. revealed another functional *IL18RAP* SNP—*rs2058660*—of which the CC/TT variants could mediate differential IL-18 responses by altering IL18RAP expression levels in neutrophils [33]. Intriguingly, this SNP also affects leprosy and Crohn’s disease susceptibility in opposite directions. Not only affecting disease susceptibility, IL18RAP expression in synovial tissues was shown to associate with treatment response in rheumatoid arthritis patients [34]. It is worth noting that gene overexpression does not always endow functional enhancement. In systemic-onset juvenile idiopathic arthritis (SJIA), an overexpression of IL18RAP in neutrophils was observed in patients with active disease [35]. However, NK cells of SJIA patients were also shown to have impaired IL-18-mediated cytotoxicity due to defective IL18RAP phosphorylation [36]. This could be one of the reasons to account for the divergent role of IL18RAP in different clinical conditions. Nevertheless, dysregulated IL18RAP expression has been observed in a number of inflammatory diseases.

In SLE research to date, there is only one published study on IL18RAP with implicated functional relevance. Neumann et al. reported an elevated IL18RAP expression in lymphocytes of MRL/*lpr* mice, which in turn was associated with heightened IL-18-induced lymphocyte proliferation and IFN-γ production [21]. Here, we demonstrate that IL18RAP expression in SLE patients—both at mRNA and protein levels—was significantly higher in circulating neutrophils when compared with healthy controls (Figure 1). Interestingly, our results suggest that a shorter isoform of IL18RAP may be expressed by neutrophils. The 52-kDa IL18RAP short isoform (UniProtKB identifier: O95256-2) has 142 amino acids missing at the N-terminal in the extracellular domain. The functional impact of this isoform is not clear. Structural and biochemical analyses showed that this region is not involved in ligand binding of the IL-18/IL-18Rα/IL18RAP ternary complex [7]. Thus, it is likely that the absence of this region would have minimal impact on IL-18 signalling. Nevertheless, the overall IL18RAP protein levels were higher in SLE neutrophils. We did not perform IL18RAP SNP analysis and whether this overexpression is related to genetic predisposition is yet to be tested. However, our data clearly show that the inflammatory milieu in patient sera can, at least in part, contribute to the IL18RAP expression upregulation. We further demonstrate that type I IFN is a potent mediator for the upregulation of IL18RAP expression in neutrophils (Figure 3). Type I IFN is known to play a central pathologic role in driving SLE development and disease progression [37]. Peripheral leukocytes of SLE patients often exhibit a highly expressed ISG signature in correlation with disease activity [26,38]. Additionally, upregulation of granulocyte-associated genes were also reported, suggesting that neutrophils also play functional roles in disease development [39]. In fact, type I IFN has been shown to prime neutrophils to undergo NETosis, a cell death pathway with the formation of NETs, which release cellular proteins and DNA that further fuel the inflammatory process in SLE patients [40]. In line with this, our findings also demonstrate another functional impact of type I IFN on neutrophil function in SLE, as highlighted by the heightened IL-18-mediated ROS production enhancement in neutrophils of SLE patients in relation to the elevated IL18RAP expression (Figure 4). On a side note, the priming or enhancement effect of IL-18 on ROS generation in neutrophils has also been shown in settings of bacterial infections, anti-neutrophil cytoplasm autoantibody (ANCA)-associated vasculitis, and rheumatoid arthritis [25,41,42].

With increasing evidence to support its pathological involvement, neutrophils from SLE patients indeed display multiple aberrations, including dysregulated cytokine and chemokine expression, defective phagocytic and antimicrobial capacity, and enhanced oxidative stress responses [43]. Notably, studies on ROS production capacity in SLE neutrophils have conflicting findings. Perozzio et al. showed that, when compared with healthy controls, neutrophils from SLE patients exhibited a higher ROS production at basal level as well as under *Staphylococcus aureus* or *Pseudomonas aeruginosa* stimulation [44]. Conversely, SLE neutrophils were shown to have decreased ROS generation after phorbol-myristate acetate (PMA) or *Escherichia coli* stimulation in an independent study [45]. The reason for such discrepancy is not clear but it could be due to intrinsic variations in different patient cohorts and stimuli. Nevertheless, a relatively recent study unravelled an essential role of mitochondrial (mt) ROS in mediating ribonucleoprotein immune complex (RNP-IC)-induced NETosis, particularly in the unique subpopulation of low density-gradient granulocytes (LDGs) that are found in SLE patients [46]. In vivo ablation of mt ROS in MRL/*lpr* mice could attenuate lupus-like symptoms, including anti-dsDNA level and renal manifestations with a concurrent reduction in NETosis [46]. Moreover, there exists increasing evidence to link nicotinamide adenine dinucleotide phosphate (NADPH) oxidase-derived ROS (e.g., induced by fMLP stimulation) with NETs formation [47]. All these studies suggest that ROS production by neutrophils could have significant pathological contribution to lupus development.

Consistent with this reasoning, our data indeed show a positive correlation between IL18RAP expression and SLE disease activity. A higher expression was also observed in patients with a history of nephritis and in patients exhibiting renal manifestations at the time of investigation (Figure 2). IL-18 has long been implicated in promoting nephritis development in SLE in the capacity of a prominent Th-1 response driver [22]. Elevated levels of IL-18 have been found in serum and urine, as well as kidney biopsies, of LN patients [13,48,49]—this could have functional impact on both circulating and kidney-infiltrating neutrophils. Potentially, higher IL18RAP expression in the infiltrating neutrophils could enhance the generation of ROS, which is known to play a major role in glomerulonephritis [50]. Taken together, our findings provide evidence to support an additional pathogenic role of IL-18 in that it may promote nephritis through the mediation of neutrophil dysfunction via the upregulated expression of IL18RAP.

## 5. Conclusions

In summary, we have revealed an overexpression of IL18RAP in neutrophils which is associated with disease activity and renal involvement in SLE patients. Type I IFN in the inflammatory milieu could cause this upregulation, which in turn leads to IL-18-mediated enhancement in ROS generation. These findings provide new insights into the role of IL-18 in contributing to the dysregulation of neutrophil function, which is now known to play a crucial role in SLE and LN development.

## Figures and Tables

**Table 1:** Demographics and clinical characteristics of study participants.

Study Group	HC (*n* = 30)	NLN (*n* = 40)	LN (*n* = 55)
Demographics			
Male: Female, *n* (%)	1:29 (3:97)	2:38 (5:95)	4:51 (7:93)
Age, median (IQR)	48 (41–53)	53 (47–61)	49 (42–59)
Serological parameters, median (IQR)			
Anti-dsDNA antibodies [IU/mL]	NA	28 (9–81)	47 (19–106)
C3 [mg/dL]	NA	83 (63–100)	79 (63–93)
C4 [mg/dL]	NA	14 (11–18)	16 (12–22)
Urea [mmol/L]	NA	5 (4–6)	5 (4–7)
Creatinine [mmol/L]	NA	64 (55–71)	69 (57–83)
Albumin [g/L]	NA	43 (40–45)	41 (37–44)
Globulin [g/L]	NA	35 (32–38)	30 (28–34)
SLEDAI-2K score	NA	2 (0–4)	4 (0–5)
Organ involvement ^a^, *n* (%)			
Central nervous system	NA	0 (0)	0 (0)
Vascular	NA	0 (0)	0 (0)
Musculoskeletal	NA	0 (0)	0 (0)
Renal	NA	0 (0)	12 (22)
Dermal	NA	6 (15)	8 (15)
Serosal	NA	0 (0)	0 (0)
Immunological	NA	25 (63)	39 (71)
Haematological	NA	7 (18)	3 (6)
Constitutional	NA	0 (0)	0 (0)
Medication, *n* (%)			
Prednisolone	NA	26 (65)	50 (91)
Azathioprine	NA	8 (20)	8 (15)
Hydroxychloroquine	NA	26 (65)	32 (58)
Mycophenolate mofetil	NA	6 (15)	36 (66)
Methotrexate	NA	1 (3)	0 (0)

^a^ Defined by the descriptors in the SLEDAI-2K score at the time of blood sample collection. C3, complement 3; C4, complement 4; HC, healthy controls; IQR, interquartile range; LN, lupus nephritis; NA, not available/not applicable; NLN, non-nephritic lupus; SLEDAI-2K, systemic lupus erythematosus disease activity index 2000.

## Data Availability

No new data outside those presented in this study were created or analyzed. Data sharing is not applicable to this article.

## Supplementary Materials

The following are available online at https://www.mdpi.com/article/10.3390/cells10050964/s1, Figure S1: Expression correlation of IL18RAP and neutrophil-associated genes in peripheral leukocytes of SLE patients, Figure S2: Neutrophils from healthy fresh blood and buffy coat have similar ability to produce ROS, Figure S3: No difference in IL-18Rα expression in neutrophils between HC and SLE patients, Figure S4: IL-18 enhances immune complex-mediated ROS production in neutrophils.

## Abbreviations

ACR	American College of Rheumatology
ANCA	anti-neutrophil cytoplasm autoantibody
C3	complement 3
C4	complement 4
DHR123	dihydrorhodamine 123
eQTL	expression quantitative trait loci
fMLP	N-formyl-methionyl-leucyl-phenylalanine
FS	fluorescence signal
GAPDH	glyceraldehyde-3-phosphate dehydrogenase
GWAS	genome-wide association studies
HC	healthy control
IFIT1	interferon induced protein with tetratricopeptide repeats 1
IFN	interferon
IFN-a	interferon-alpha
IFN-g	interferon-gamma
IL-18	interleukin-18
IL-18R	interleukin-18 receptor
IL-18R1/IL-18Ra	interleukin-18 receptor alpha
IL18RAP/IL-18Rb	interleukin-18 receptor accessory protein/interleukin-18 receptor beta
IL-1R	interleukin-1 receptor
IQR	interquartile range
ISGs	interferon-stimulated genes
LDGs	low density-gradient granulocytes
LN	lupus nephritis
MAPK pathway	mitogen-activated protein kinase pathway
mtROS	mitochondrial reactive oxygen species
MyD88	myeloid differentiation primary response protein 88
NA	not available/not applicable
NADPH	nicotinamide adenine dinucleotide phosphate
NETs	neutrophil extracellular traps
NF-kB	nuclear factor kappa-light-chain-enhancer of activated B cells
NKcells	natural killer cells
NLN	non-nephritic lupus
PBMCs	peripheral blood mononuclear cells
PMA	phorbol-myristate acetate
qRT-PCR	real-time quantitative reverse transcription polymerase chain reaction
rhIL-18	recombinant human interleukin-18
RNP-IC	ribonucleoprotein immune complex
ROS	reactive oxygen species
RQ	relative quantity
SJIA	systemic-onset juvenile idiopathic arthritis
SLE	systemic lupus erythematosus
SLEDAI-2K	systemic lupus erythematosus disease activity index 2000
SLICC	Systemic Lupus International Collaborating Clinics
SNP	single nucleotide polymorphism
Th-1 cells	type 1 T helper cells
TIR	toll-interleukin-1 receptor
TLR	toll-like receptor
