# Supplementary material for: Elevated Interleukin-18 Receptor Accessory Protein Mediates Enhancement in Reactive Oxygen Species Production in Neutrophils of Systemic Lupus Erythematosus Patients
Source: Cells. 2021 Apr 21;10(5):964. doi: 10.3390/cells10050964 (PMC8143138; doi:10.3390/cells10050964)
Supplement: Supplementary file 1 [file cells-10-00964-s001.zip › cells-1174789-supplementary.pdf]

cells MDPI

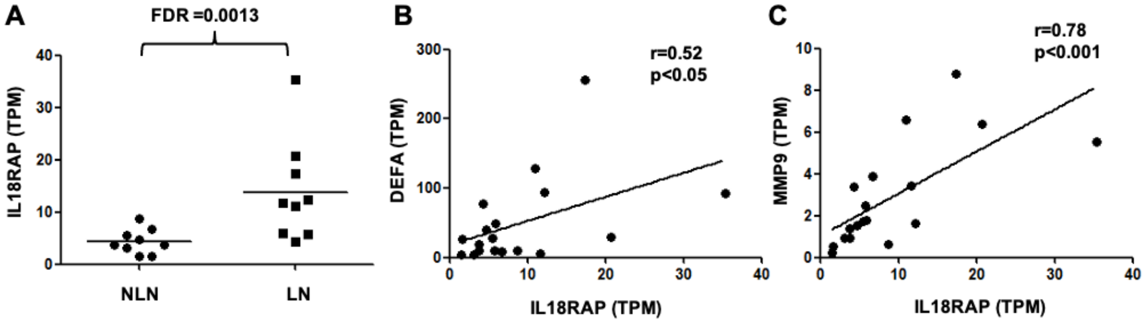

Supplementary Figure S1. Expression correlation of IL18RAP and neutrophil-associated genes in peripheral leukocytes of SLE patients. Transcriptome analysis on peripheral blood leukocytes from SLE patients with (LN) or without (NLN) biopsy-confirmed nephritis (n=9 in each group) was performed by RNA sequencing. Differential genes were identified using the EBSeq version 1.10.0 software (A) Expression of IL18RAP was compared between LN and NLN. Expression correlation between (B) α-defensin (DEFA), (C) matrix metallopeptidase 9 (MMP9) and IL18RAP were analysed by Spearman's rank-order test, r=correlation coefficient. Linear regression line was shown. FDR: false detection rate. TPM: transcripts per million.

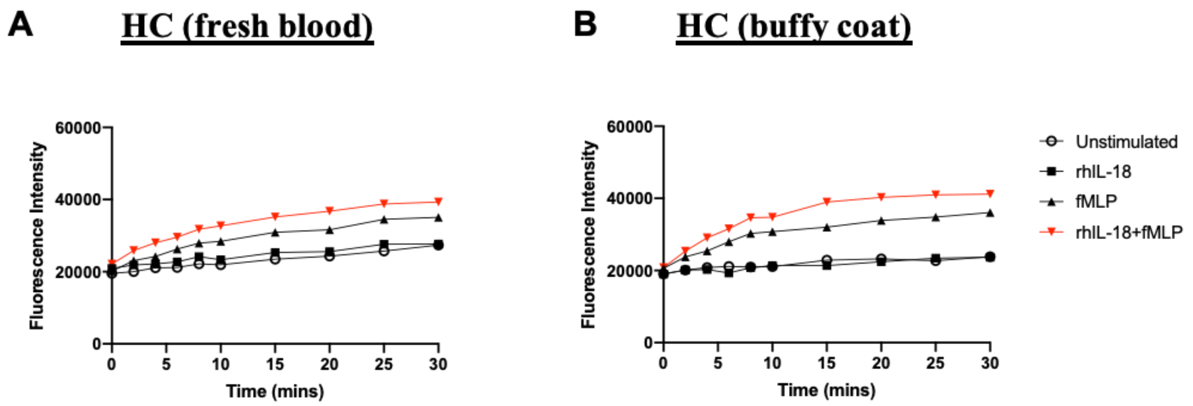

Supplementary Figure S2. Neutrophils from healthy fresh blood and buffy coat have similar ability to produce ROS. Kinetics of ROS generation in neutrophils from (**A**) fresh blood (**B**) buffy coat upon treatment with or without rhIL-18 (100 ng/mL) and fMLP (100 nM) stimulation. Representative plots of two independent experiments are shown.

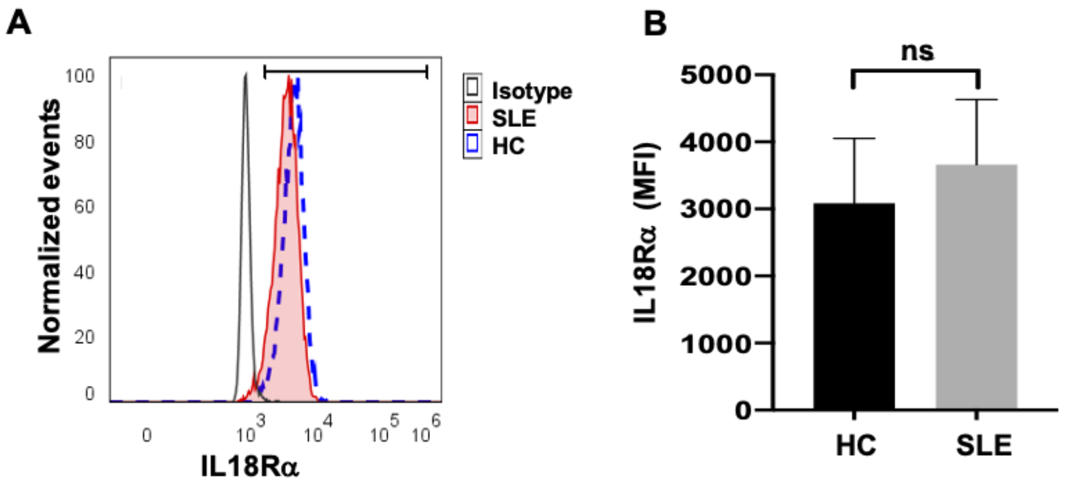

Supplementary Figure S3. No difference in IL-18Rα expression in neutrophils between HC and SLE patients. Peripheral leukocytes were stained with anti-IL18α antibody (Clone H44, Biolegend, USA) or mouse IgG1 isotype control antibody and analysed by Flow Cytometry. (**A**) Representative histogram plot showing IL-18Rα expression in gated neutrophils from healthy control (HC) and SLE patients. (**B**) Summary chart comparing IL-18Rα expression in neutrophils between HC (n=4) and SLE patients (n=6) with no statistical significance (ns) by Mann-Whitney *U* test. MFI: mean florescence intensity.

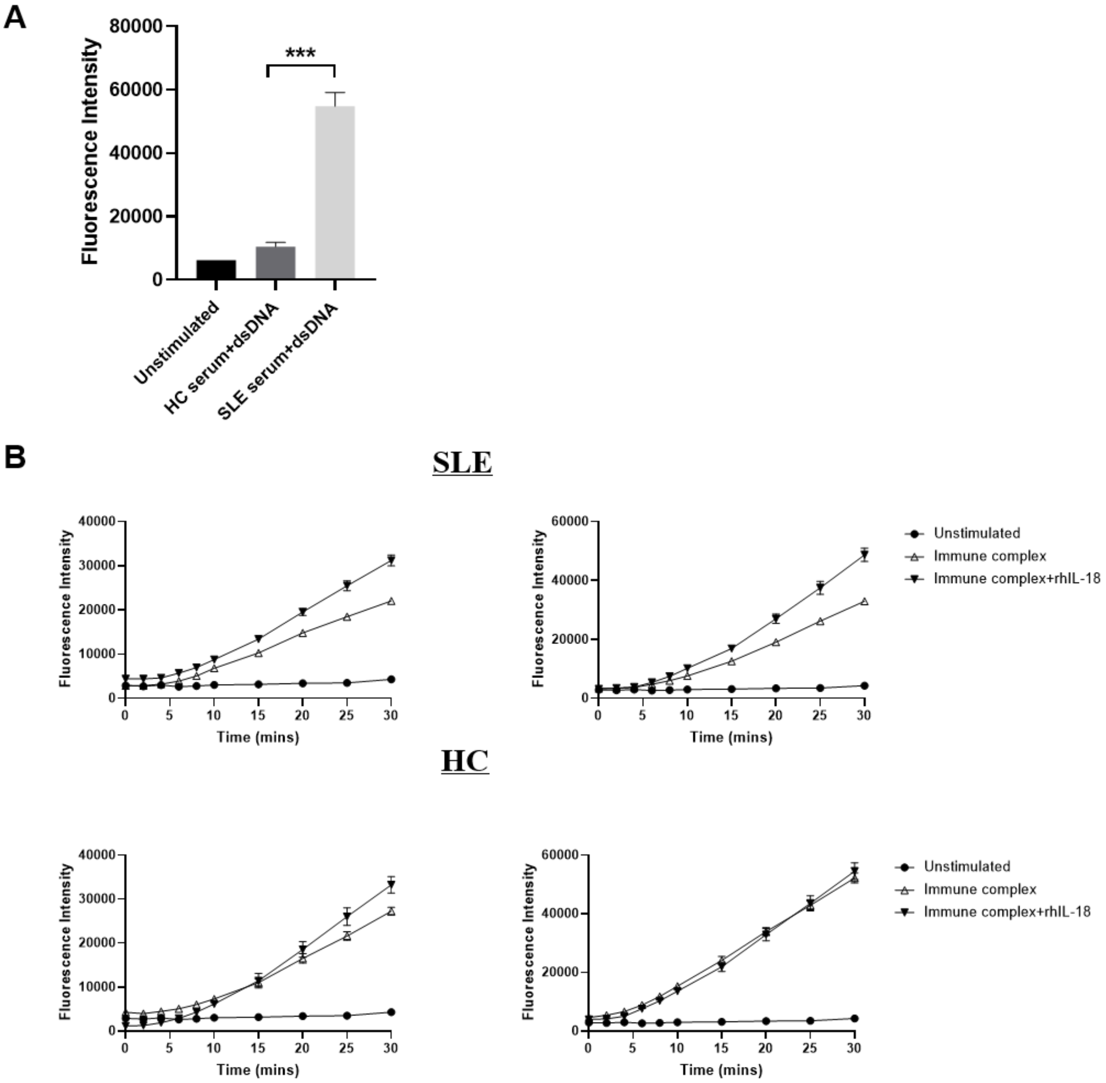

Supplementary Figure S4. IL-18 enhances immune complexes-mediated ROS production in neutrophils. (**A**) Summary chart showing plate-bound immune complexes formed by double-strand DNA (dsDNA) and sera from SLE patients (n=4) but not sera from HC (n=3) can induce ROS generation in neutrophils at 30-minute post stimulation. ***p<0.001 by unpaired t test. (**B**) Representative plots showing kinetics of ROS production in neutrophils from two SLE patients and two heathy controls (HC) upon treatment with or without rhIL-18 (100 ng/mL) and plate-bound anti-dsDNA immune complexes stimulation.

Method: Formation of plate-bound anti-dsDNA immune complexes: 20 μg/mL dsDNA was coated in high-binding 96-well plate overnight. After washing with PBS, wells were blocked with 2% BSA in PBS for 1 hour at room temperature following 2 hours incubation with sera from HC or SLE patients. Finally, immune complexes-coated wells were washed with PBS before adding with neutrophils to detect ROS induction as described in the Materials and Methods section in main text.
